# Enhanced Bone Formation in Segmental Defect Healing Using 3D Printed Scaffolds Containing Bone Marrow Stromal Cells and Small Molecules Targeting Chondrogenesis and Osteogenesis

**DOI:** 10.3390/biomedicines14010227

**Published:** 2026-01-20

**Authors:** Charles H. Rundle, Sheila Pourteymoor, Enoch Lai, Chandrasekhar Kesavan, Subburaman Mohan

**Affiliations:** 1 Musculoskeletal Disease Center, Veterans Affairs Loma Linda Healthcare System, Loma Linda, CA 92357, USAchandrasekhar.kesavan@va.gov (C.K.); 2 Department of Medicine, Loma Linda University, Loma Linda, CA 92350, USA; 3 Department of Gastroenterology, Veterans Affairs Loma Linda Healthcare System, Loma Linda, CA 92357, USA; 4 Department of Orthopedic Surgery, Loma Linda University, Loma Linda, CA 92350, USA; 5 Department of Basic Sciences, Loma Linda University, Loma Linda, CA 92350, USA

**Keywords:** segmental defect, 3D-printed implant, sequential therapy, chondrogenesis, angiogenesis

## Abstract

**Background/Objectives:** Nonunion bone healing results from a critical size defect that fails to bridge a bone injury to produce bony union. Novel approaches are critical for refining therapy in clinically challenging bone injuries, but the complex and coordinated nature of fracture callus tissue development requires study outside of the simple closed murine fracture model. **Methods:** We have utilized a three-dimensional printing approach to develop a scaffold construct with layers designed to sequentially release small molecule therapy within the tissues of a murine endochondral segmental defect to augment different mechanisms of fracture repair during critical stages of nonunion bone healing. Initially, a sonic hedgehog (SHH) agonist is released from a fibrin layer to promote chondrogenesis. A prolyl-hydroxylase domain (PHD)2 inhibitor is subsequently released from a β-tricalcium phosphate (β-TCP) layer to promote hypoxia-inducible factor (HIF)-1α regulation of angiogenesis. This sequential approach to therapy delivery is assisted by the inclusion of bone marrow stromal cells (BMSCs) to increase the cell substrate available for the small molecule therapy. **Results:** Immunohistochemistry of fracture callus tissue revealed increased expression of PTCH1 and HIF1α, targets of hedgehog and hypoxia signaling pathways, respectively, in the SAG21k/IOX2-treated mice compared to vehicle control. MicroCT and histology analyses showed increased bone in the fracture callus of mice that received therapy compared to control vehicle scaffolds. **Conclusions:** While our findings establish feasibility for the use of BMSCs and small molecules in the fibrin gel/β-TCP scaffolds to promote new bone formation for segmental defect healing, further optimization of these approaches is required to develop a fracture callus capable of completing bony union in a large defect.

## 1. Introduction

Of the approximately 16 million fractures that occur annually in the United States, 10% to 20% proceed to severely delayed or nonunion healing [1]. Impaired healing could result from the severity of the injury or from pathophysiological conditions affecting bone formation and resorption, such as diabetes [2,3] or age-related complications that impair otherwise routine bone repair [4]. Among femoral and tibial fractures in the general population, the return to normal physical function without pain can be difficult even for routine healing in healthy individuals and delayed and nonunion healing outcomes can be significantly worse [5].

The development of therapeutic approaches for clinical applications to critical size defects is therefore an important objective. These defects occur through the severity of bone injury, and the size of the injury in fracture repair has a huge effect on the progress of healing. Critical size defects do not heal without intervention and may even result from impaired healing of otherwise routine bone injuries in individuals compromised by numerous physiological conditions [6,7]. These situations often require surgical resection, and are typically still very slow to heal, progressing to severely delayed union or even nonunion [8]. To overcome this impairment with therapeutic intervention, it is critical to understand the molecular pathways that regulate closed fracture repair but that are more impaired in segmental defect healing [9].

Traditional approaches to studies in endochondral bone fracture repair have utilized animal models, usually adaptations of the hindlimb closed fracture model in the rat or mouse [10,11,12,13,14]. These models have provided valuable information on bone fracture repair, but the development of therapeutic options is limited in models where bone healing is not compromised by the severity of the injury or a complicating physiological condition. The development of therapy has therefore progressed from simple closed fracture models with efficient healing to closed fractures with compromised healing or to more severe bone injuries where the normal events in bony callus development are defective and the treatment of atrophic, aseptic nonunion healing must be promoted using novel approaches (reviewed in [15]).

We have previously developed a segmental defect model of murine femoral bone injury and used it to identify deficiencies in defect repair [16]. This model fails to heal when a 2.0 mm width gap is introduced into the femur, in agreement with other segmental defect approaches [15]. During healing, the defect becomes occupied with non-periosteal fibrous tissue that lacks bone formation potential and simply produces fibrous scar tissue between the proximal and distal edges of bone in the defect, a definitive characteristic of nonunion bone healing. Using this model, we have identified a major impediment to bone repair as an impairment of soft callus development from the periosteum that fails to provide a sufficient tissue substrate for sufficient cartilage development and subsequent bone formation [17,18,19,20].

The development of 3D-printing capabilities for the production of implants using specific materials has assisted the study of segmental defect bone repair (reviewed in [21,22,23]). The implanted 3D-printed scaffold can provide a physical structure within the defect gap that can support the development of a bony callus and mediate healing of the defect, a space devoid of tissues with chondrogenic or osteogenic potential. The scaffold can further enhance bone repair through the controlled release of therapeutic agents incorporated into the matrix materials to the surrounding tissues or facilitate tissue development on the scaffold itself.

Our previous attempts to promote bone formation within the critical size defects have used different applications of therapeutic mediators designed to sequentially promote chondrogenesis and angiogenesis in the defect gap and recapitulate normal fracture callus development. The “Smoothened” (SMO) agonist SAG21k promote was chosen to promote chondrogenesis and cartilage development [24,25]. The IOX-2 inhibitor of the prolyl hydroxylase domain (PHD)-2 inhibitor of hypoxia-inducible factor (HIF)-1α was then applied to promote angiogenesis and bone formation in the defect cartilage [26,27]. The first study applied these mediators through systemic injections at different times during segmental defect healing [28] and met with limited success. A subsequent study emphasized the local application and sequential release of these mediators from 3D-printed scaffolds that were implanted into the defect during the segmental defect surgery. In this case, SAG21k was incorporated into an outer fibrin layer to promote chondrogenesis, and IOX-2 was incorporated into an inner β-tricalcium phosphate (β-TCP) layer of the scaffold to be released and promote angiogenesis when the outer fibrin layer was degraded [29]. This local sequential therapy approach also met with limited success. We concluded that it is necessary to also incorporate cells with chondrogenic and osteogenic potential into the implanted scaffold that provide a target capable of responding to the initial chondrogenic stimulus and subsequent angiogenic promotion by the small molecule therapy with the sequential development of cartilage and bone. Bone marrow stromal cells (BMSCs) have multilineage differentiation potential and are used extensively in skeletal repair (ref. [30], reviewed in [31]). These cells could potentially recapitulate periosteal cell functions that mediate normal closed fracture healing [32,33].

We therefore modified the application of the local small molecule therapy that we previously applied to 3D-printed implants within the segmental defect. We hypothesized that a cell substrate with osteochondrogenic potential applied to the implant scaffold was necessary for the therapeutic molecules to produce bone. These cells should provide a substrate for either the direct differentiation to osteoblasts or the formation of chondrogenic cells that either become osteoblasts through transdifferentiation [34,35] or mediate osteoblast development following cartilage resorption [12]. In this case, we applied exogenous BMSCs onto the fibrin layer after printing that had incorporated the “Smoothened” agonist SAG21k into this layer. This adaptation aimed to activate hedgehog signaling and promote chondrogenesis from those BMSCs, to be followed by the release of IOX2 from the β-TCP layer, which promotes angiogenesis and osteogenesis within the defect cartilage and ultimately promotes bone formation.

## 2. Materials and Methods

### 2.1. Implant Preparation and 3D Printing

A two-layer scaffold was printed for the characterization of cell functions in vitro and application of the therapy to the segmental defect in vivo. To prepare a C-shape scaffold for implantation at the defect site, a rectangular 3D design measuring 5 mm × 5 mm × 1 mm was created using online Tinkercad v1.4 software (Autodesk, San Francisco, CA, USA). The design was then sliced using a slicing software with an 80% infill rate and the file was saved in G-code format for printing.

All procedures were performed under a bio-safety hood in a sterile environment. β-TCP and fibrin gel scaffold preparations were purchased from CELLINK (Gothenburg, Sweden). Prior to printing, the β-TCP and fibrin gel samples were warmed to room temperature and then mixed with appropriate concentrations of IOX2 (for β-TCP) and SAG21K (for fibrin). Each therapeutic loaded cartridge was connected to a 400-micron nozzle on the “INKREDIBLE+ 3D” bioprinter (CELLINK).

The fibrin layer was printed first at a pressure of 35 psi, followed by the β-TCP at a pressure of 75 to 100 psi. To print the fibrin gel, two printing cartridges were prepared, one that contained fibrinogen solution (fibrinogen + aproteinin) and the other that contained thrombin solution (thrombin, CaCl_2_ + heparin). Fibrinogen solution + SAG21k (2 mg/mL) or vehicle was printed on a 60 mm tissue culture dish and thrombin solution + SAG21k (2 mg/mL) or DMSO vehicle was printed directly onto the fibrinogen solution with a temperature-controlled printhead from CELLINK as per manufacturer instructions. The fibrinogen solution was allowed to polymerize at room temperature for 30 min. β-TCP (BONE Bioink preparation from CELLINK) containing IOX2 (10 mg/mL) or DMSO vehicle was printed and cured for 60 min under UV light as per manufacturer instructions for the INKREDIBLE + 3D printer.

After printing, the scaffold was cut longitudinally to approximately 4 mm length × 2 mm width × 1 mm thickness and carefully rolled into a C-shape. The C-shaped scaffold was then cured under 365 nM UV light and stored in PBS-soaked gauze to maintain adequate moisture until BMSC application and implantation.

BMSCs were applied and allowed to attach to the printed scaffold on the day before the surgical procedures and maintained in αMEM with 10% fetal bovine serum overnight. The 3D-printed scaffold with BMSCs was rinsed with PBS for implantation around the intramedullary rod in the defect gap during segmental defect surgery. The final scaffold size for implantation into the segmental defect was approximately 4 mm length × 2 mm width × 1 mm thickness.

### 2.2. Therapy Release from the Scaffold In Vitro

The cumulative release profile of SAG21k and IOX2 from the scaffold material was determined in culture over a three-week period. The fibrin gel/βTCP scaffolds containing SAG21k, IOX2, or DMSO vehicle were incubated in PBS at room temperature and an aliquot was removed every 2 to 3 days for measurement of drug concentration by optical density at 230 nm.

### 2.3. Bone Marrow Stromal Cell Preparation

Bone marrow stromal cells (BMSCs) were isolated from the hindlimbs of 12-week-old male C57BL/6 mice. Hindlimb bones were aseptically dissected from individuals, and cells were flushed from the intramedullary space of the hindlimb bones, pelleted, and propagated in complete αMEM media without therapy for one week prior to use at Passage 0. Adherent cells were assumed to be BMSCs.

### 2.4. BMSC Response to Small Molecule Therapy In Vitro

The response of the cells to the small molecule therapy that we proposed for the sequential promotion of chondrogenesis and angiogenesis was determined in culture. In this case, the isolated adherent cells were cultured to 50% confluence in αMEM with 10% fetal bovine serum, changed to serum-free media containing 0.1% BSA for 24 h and treated for three days with (1) 10 nM SAG21k, (2) 20 μM IOX-2, (3) a combination of SAG21k and IOX-2, or (4) a DMSO vehicle control.

Total RNA was then isolated from the BMSCs and the expression of markers of chondrogenesis and osteogenesis, as well as targets of the sonic hedgehog and HIF-1α pathways, were examined by real-time RT-PCR with gene-specific primers by real time RT-qPCR. Gene expression was normalized to expression of the peptidylprolyl isomerase A (*Ppia*, cyclophilin-A) housekeeping gene. Genes and primer sequences are presented in Table S1. Changes in gene expression in culture were determined from the change in cycle number in gene expression relative to the housekeeping gene (ΔCt) in response to SAG21k and IOX-2, individually or in combination, compared to culture with the vehicle (ΔΔCt). Gene expression is presented as the fold-change calculated from the ΔΔCt.

### 2.5. BMSC Survival on the Implant In Vitro

The survival of the BMSCs on the fibrin/β-TCP implant materials was determined by longitudinal culture of BMSCs derived from Td-Tomato floxed mice (Jackson Laboratories, Bar Harbor, ME, USA) that were infected with adenoviral vectors expressing Cre recombinase to induce expression of fluorescent tomato red marker protein. A total of 2 × 10^5^ BMSCs expressing the tomato red fluorescent marker were then applied to the fibrin-β-TCP scaffold. The scaffolds containing BMSCs expressing tomato red protein were incubated in αMEM containing 10% fetal bovine serum for 21 days. Cells expressing tomato red fluorescent marker were evaluated weekly in multiple 1 mm^2^ areas to determine whether they retained viability when implanted in the scaffold constructs.

### 2.6. Segmental Defect Surgery

All procedures involving animals were reviewed and approved by the Institutional Animal Care and Use Committee. All mice used in the segmental defect surgery were 12- to 13-week-old male C57BL/6 mice. The segmental defect model has previously been described [16].

Mice received a short-term subcutaneous application of 60 μg/kg Buprenorphine for surgery. Inhalation anesthesia of 3% isoflurane, 0.5 L/min oxygen was delivered through a Bains non-rebreathing circuit. A skin incision to the lateral upper hindlimb exposed the biceps femoris and vastus lateralis muscle groups, which were then separated to expose the femoral shaft. A 0.5 mm diameter threaded rod with plastic spacers was inserted into the intramedullary cavity following the production of a midshaft defect of approximately 2.0 mm width. Plastic spacers on either end of the rod were positioned at the proximal and distal ends of the cavity to adjust and maintain the 2.0 mm spacing between the proximal and distal ends of the femur.

**Figure 1:**
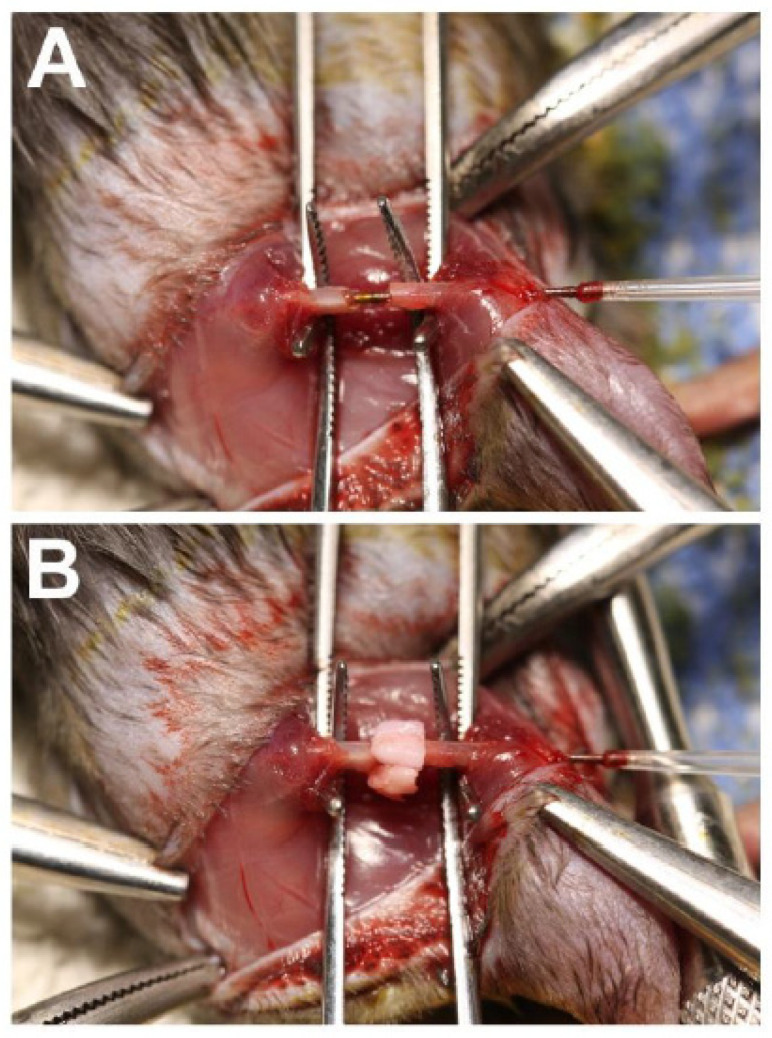
Surgical approach for the implantation of the construct in the femoral segmental defect. (**A**) The femoral defect is stabilized with plastic spacers positioned on an intramedullary threaded rod that maintain the defect spacing at approximately 2.0 mm. (**B**) The scaffold is wrapped around the rod and secured within the defect with the β-TCP layer inside the outer fibrin layer.

Syngeneic bone marrow stromal cells (BMSCs) were isolated from 12- to 13-week-old male C57BL/6J mice and approximately 1 × 10^5^ cells applied to the fibrin surface of two-layer 3D-printed scaffolds with SAG21k (0.04 mg/mL) in the fibrin layer and IOX2 (3.0 mg/mL) without BMSCs in the β-TCP layer. The SAG21k and IOX2 small molecules were incorporated into their respective fibrin and β-TCP layers of the 3D-printed scaffold and the BMSCs applied to the scaffold on the day before the segmental defect surgery. During the segmental defect surgery, the scaffold was implanted into the segmental defect gap by wrapping it around the surgically implanted rod so that the fibrin-BMSC/SAG21k layer with the BMSCs formed an outer circumference that surrounded the inner β-TCP/IOX2 layer. The implant was secured with a drop of 3M Scotch-Weld Super Glue. Figure 1 shows the approach to secure the implant within the segmental defect. A group of eight animals with the scaffold/BMSC/SAG21k-IOX2 therapy was compared with six control animals with the scaffold/BMSC/no therapy.

The muscle groups and skin were then sutured with 4.0 Vicryl and the production of a femoral segmental defect confirmed by X-ray examination. When recovery was imminent, longer-term analgesia was achieved using Ethiqa XR Buprenorphine (Fidelis Animal Health, New Brunswick, NJ, USA), applied subcutaneously at 3.25 mg/kg. The success of the surgery was confirmed by X-ray examination of the hindquarters. The animal was allowed to recover with unrestricted movement. All mice were monitored daily for satisfactory movement and comfort.

### 2.7. Analysis of Healing

The animals were monitored for post-surgery healing in vivo by periodic X-ray examination under isoflurane anesthesia. This approach also confirmed that defect compression due to apparatus failure was avoided and that the 2 mm critical size defect size set during surgery to prevent healing was maintained.

Harvest was performed at eight weeks post-surgery, a time determined from previous experience with the segmental defect model, in which delayed healing had become nonunion healing and when the efficacy of our therapy could be best evaluated. The entire femur was harvested and fixed in 10% formalin for 3 days. The apparatus was removed through the femoral condyle by simply unwinding the threaded rod through the bone; any plastic tubing from the apparatus that remained in the intramedullary cavity was largely inert with respect to subsequent radiology and histology. Immunohistochemical analysis for the targets of the PHD2 pathway was also performed at 8 weeks post-surgery. Samples analyzed for SAG21k gene targets of the sonic hedgehog (SHH) pathway by immunohistochemistry were harvested at 21 days post-surgery, estimated as the time required for cartilage development in the segmental defect model.

MicroCT analysis was performed using Scanco vivaCT40 instrumentation and software (Scanco Medical AG, Zurich, Switzerland). Scans of the entire defect included the cortical bone at the defect edges and were performed at 55 kV and 145 μA. Analysis was performed using a two-threshold density approach that separated the bone scans into density thresholds of 250 to 570 mg HA/cm^3^, characteristic of lower-density woven callus bone, and 570 to 1000 mg HA/cm^3^, characteristic of higher-density cortical bone. The bone volume and partial bone volume were compared in defects receiving the therapeutic implants and control implants.

Femurs were then decalcified in EDTA, paraffin-embedded, and sectioned at 5 μm thickness for standard histology staining by Trichrome for collagen or for immunohistochemistry for the small molecule pathway targets. Sections were examined for callus bone and cartilage formation using an Evident BX-60 microscope with a DP75 camera and “cellSens Entry 4.3” software (Evident, Bethlehem, PA, USA). 

### 2.8. Immunohistochemistry for SAG21k and IOX2 Targets

Fluorescent immunohistochemistry was performed on defect samples harvested at the post-surgery times when cartilage and bone formation would be expected in this defect model. At 3 weeks, SHH promotion of chondrogenesis in response to SAG21k was expected, and at 8 weeks, HIF-1α expression in response to the promotion of angiogenesis through IOX2 inhibition of PHD2 should be evident. The primary antibodies (summarized in Table S2) were the rabbit polyclonal antibodies anti-PTCH1 (nb200-118) and anti-HIF-1a (nb100-134, validated by the supplier through biological assays). Antigen retrieval was performed using “UNI-TRIEVE” (Innovex Biosciences, Richmond, CA, USA) and sections incubated overnight in 1:50 dilutions of the primary antibody. Specific primary antibody staining was compared to sections incubated with rabbit IgG isotype controls. Primary antibody binding was detected with a “Dylight 488” fluorescent kit with a horse anti-rabbit antibody (Vector Labs, Newark, CA, USA) incubated for one hour. Sections were mounted with EMS FluoroGel (Electron Microscopy Sciences, Hatfield, PA, USA) and examined under fluorescent illumination with an Evident BX-60 microscope.

### 2.9. Statistics

Results are expressed as mean ± standard error of the mean (SEM). Statistics were performed by one-tailed *t*-test comparison of small molecule therapy treatments to vehicle treatments and results were deemed significant at *p* = 0.05.

## 3. Results

### 3.1. BMSC Gene Expression in Response to Small Molecule Therapy In Vitro

**Figure 2:**
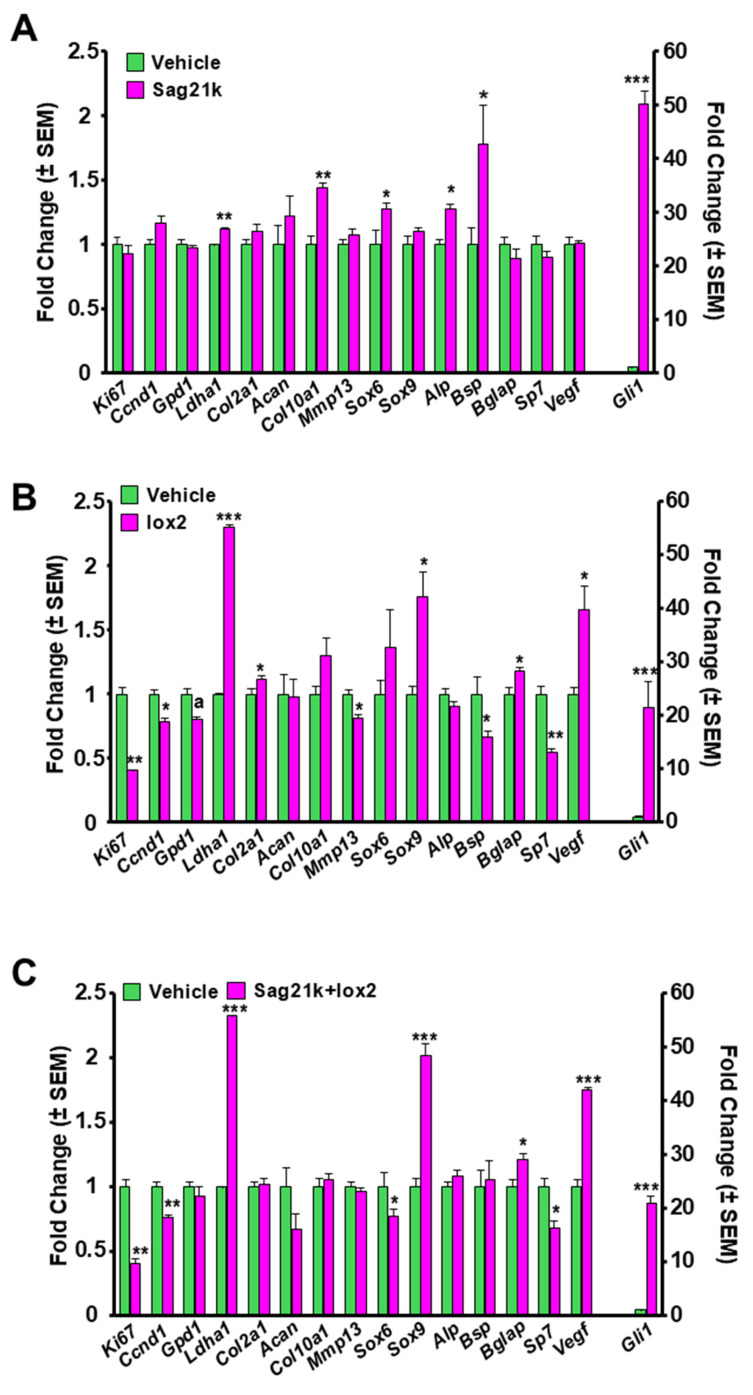
In vitro response of BMSCs to small molecule therapy. Real-time RT-qPCR measured changes in gene expression following BMSC culture with (**A**) SAG21K, (**B**) IOX2, and (**C**) a combination of SAG21k and IOX2. The genes examined represent targets of SMO and PHD2 signaling pathways, as well as cellular processes including proliferation, differentiation, glycolysis, and angiogenesis. * *p* < 0.05, ** *p* < 0.01, *** *p* < 0.001 vs. vehicle treated control (n = 3–4 per group).

Treatment of the cells with SAG21K and IOX2 in monolayer culture produced an increase in the hedgehog and hypoxia signaling responsive genes, respectively. Combined treatment with both SAG21K and IOX2 promoted expression of both hedgehog- and HIF1α-responsive genes, indicating that the cells proposed for therapy in vivo were responsive to the therapeutic small molecules in vitro and suitable for therapeutic application to the segmental defect (Figure 2).

*Gli1* expression was dramatically increased in SAG21k, IOX2, and SAG21k/IOX2 cultures, indicating activation of Shh signaling in each case, but especially in SAG21k-treated cultures. BMSC proliferation, as determined by *CyclinD1* and *Ki67* expression, was reduced in IOX2- and IOX2/SAG21K-treated cultures but not in SAG21k-treated cultures. The increase in *Ldha1* expression in IOX2-treated cultures is consistent with the known effect of this agent in promoting glycolysis. SAG21k increased the expression of *Sox6*, *Alp*, and *Col10*, suggesting a role in chondrocyte differentiation, and possibly osteoblast differentiation. *Bsp* was also increased in expression, consistent with mineralization in bone formation. While *Col2* expression was not increased by treatment with SAG21k (Figure 2A), IOX2 treatment caused a significant increase in *Sox9* expression and a modest increase in *Col2* expression in BMSCs, suggesting a role in differentiation of BMSCs towards pre-hypertrophic chondrocytes. *Acan* was not increased in expression, suggesting that it was not regulated in chondrocyte formation. Alternatively, it is possible that monolayer culture conditions limited the chondrocyte response, and that it might have been increased in a high-density BMSC culture conducive to chondrocyte development or in a cell type with greater chondrogenic potential, such as periosteal cells. *Vegf* expression was also increased in response to IOX2, suggesting a function in angiogenesis mediated through HIF-1α (Figure 2B). The large increase in *Ldha1* expression in response to IOX2 and SAG21k/IOX2 was consistent with glycogenesis driving BMSC differentiation; with the increase in *Vegf* expression, it would suggest an increased differentiation of endothelial cells (Figure 2C). Surprisingly, *Sp7* expression, important in early osteoblast development, was down-regulated in response to IOX2 and combined SAG21k/IOX treatments, suggesting an inhibitory effect. Similarly, the large increase in *Sox9* expression in response to IOX2 and combined SAG21k/IOX2 treatment suggests that IOX2 has a function in cartilage development.

### 3.2. Therapy Release Profile from the Scaffold In Vitro

**Figure 3:**
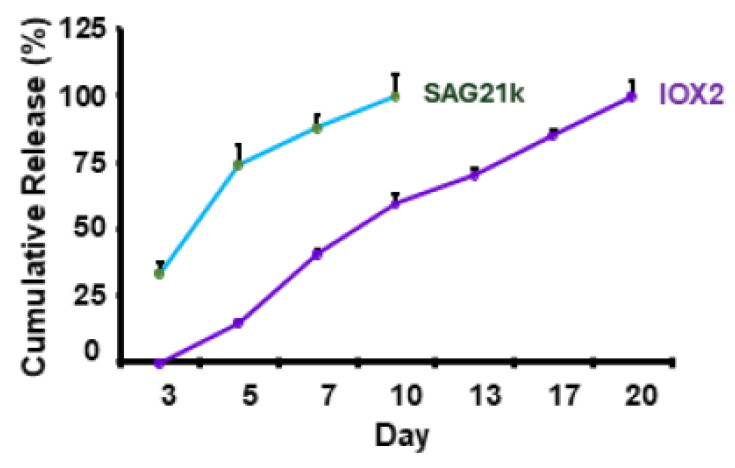
Cumulative in vitro release of SAG21k from fibrin gel–SAG21k and IOX2 from βTCP-IOX2 3D-printed scaffolds.

Figure 3 shows cumulative release of SAG21k from the fibrin gel–SAG21k scaffold and IOX2 release from the βTCP-IOX2 scaffold. The burst release of SAG21k and sustained release of IOX2 are consistent with what is known about the degradation rates of fibrin gel and βTCP. SAG21k displayed a more immediate release from the scaffold in culture, reaching 100% in 10 days, while the IOX2 release was more sustained, reaching complete release at approximately three weeks. These release profiles provide therapy availability for the sequential promotion of chondrogenesis by SAG21k and osteogenesis by IOX2 within the defect.

### 3.3. BMSC Survival on Scaffold Implant In Vitro

**Figure 4:**
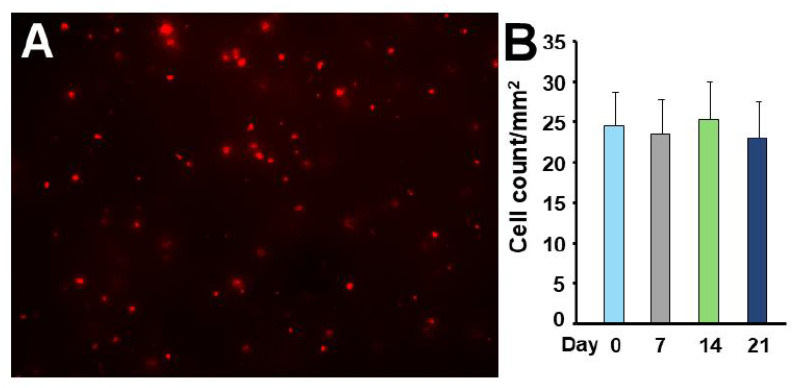
BMSC survival was confirmed on the 3D-printed scaffold matrix. (**A**) Tomato expression is visualized by Ad-Cre driven expression of TdTomato in BMSCs applied to the matrix. Scale bar = 100 μm. (**B**) TdTomato-expressing BMSCs were counted over the entire matrix surface for three weeks in culture.

Bone marrow stromal cells expressing the tomato red fluorescent marker (Figure 4A) were cultured on the fibrin/β-TCP implant. The survival of the BMSCs on the fibrin/β-TCP implant material was determined by the numbers of BMSCs that expressed the fluorescent tomato red marker. Cells expressing this fluorescent marker were visible at 21 days (Figure 4B), indicating that they retained the viability present at seeding of the implant materials until at least this time. These cells were therefore suitable for application to the defect for in vivo therapy.

### 3.4. In Vivo X-Ray Analysis of Segmental Defect Therapy

**Figure 5:**
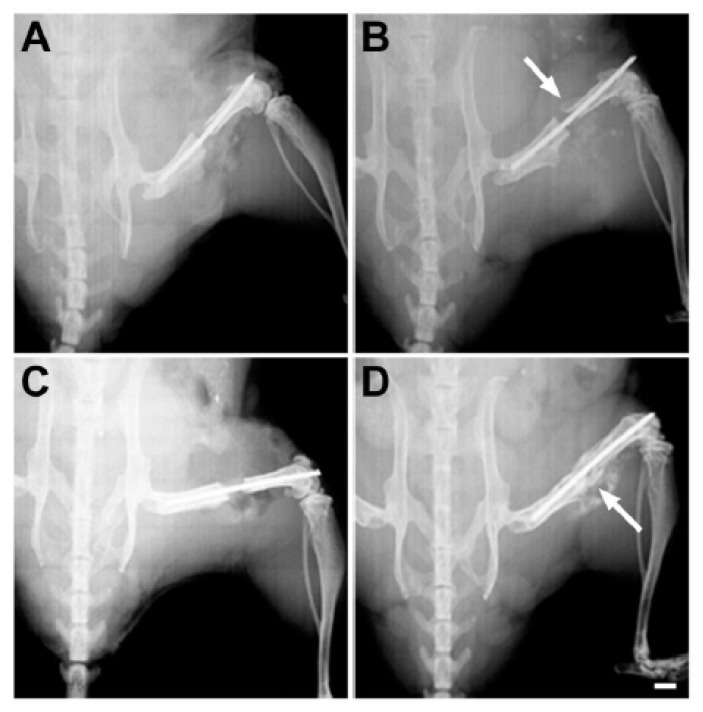
Bone formation in response to therapy was monitored by in vivo X-ray examination of the segmental defect. (**A**) The defect with the scaffold/BMSC control at surgery. (**B**) A small hard callus was often visible in defects with this control, as seen on the distal side of the defect at 8 weeks post-surgery (arrow), but bone formation had not proceeded beyond this stage. (**C**) The scaffold in the scaffold/BMSC/SAG21k-IOX2 therapy was often easily visible at surgery. (**D**) Mineralized tissue was obvious in the therapy defect at 8 weeks post-surgery (arrow). Scale bar = 1 mm.

Weekly in vivo X-ray examination monitored bone formation in segmental defects receiving the vehicle or the therapy. Representative X-ray images at surgery (Figure 5A) and at 8 weeks post-surgery (Figure 5B) confirmed that the defect gap introduced at surgery was maintained in mice receiving scaffolds that contained vehicle with no cells. Radio-dense material that is assumed to be mineralized bone was observed in the gap by 5 weeks post-surgery in mice that received implants containing BMSCs and SAG21k/IOX2 therapy and persisted to harvest at 8 weeks (Figure 5C,D). In comparison, there was much less radio-dense material in the scaffold/BMSCs control tissues.

### 3.5. Immunohistochemistry of Pathway Targets of SAG21k and IOX2 Expression

**Figure 6:**
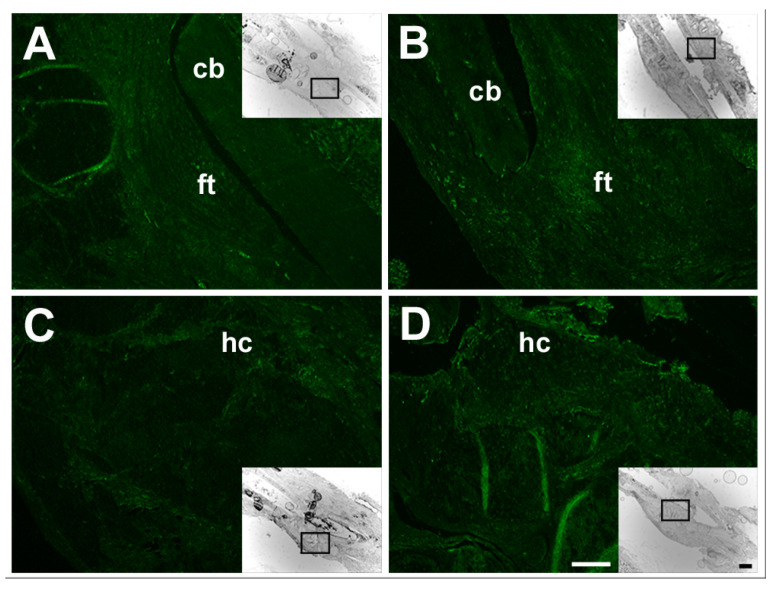
Immunohistochemistry localized the target genes of SAG21k and IOX2 in the defect tissues of the combination scaffold/BMSC/SAG21k-IOX2 therapy. (**A**) PTCH1 expression was visible in the scaffold/BMSC control fibrous tissues at 3 weeks post-surgery. (**B**) PTCH1 expression appeared augmented in scaffold/BMSC/SAG21k-IOX2 defect fibrous tissues at 3 weeks post-surgery. (**C**) HIF-1α expression was barely visible in scaffold/BMSC control defect tissues at 8 weeks post-surgery. (**D**) HIF-1α expression was visible in the hard callus of the scaffold/BMSC/SAG21k-IOX2 defect tissues at 8 weeks post-surgery. Insets identify the high magnifications of defect callus tissues. Scale bar = 250 μm, 1 mm in inset. cb, cortical bone; ft, fibrous tissue; hc, hard callus.

An examination of the defect tissues for their response to each small molecule therapy revealed an increased expression of pathway intermediates that suggest they are targets of SAG21k or IOX2 regulation (Figure 6).

At 3 weeks post-surgery, when SMO pathway functions would be expected to augment defect cartilage, the expression of PTCH1 was more abundant in the soft tissues of the defect callus (Figure 6A,B). These results suggest that cartilage development in the segmental defect is responsive to SAG21k therapy. Immunohistochemistry for gene expression consistent with PHD2 pathway regulation demonstrated an increase in HIF-1α protein level in the callus tissues in response to SAG21k/IOX2 therapy at 8 weeks post-surgery (Figure 6C,D), suggesting that HIF-1α-mediated angiogenesis necessary for bone formation was augmented.

### 3.6. MicroCT Analysis of Defect Bone Formation

**Figure 7:**
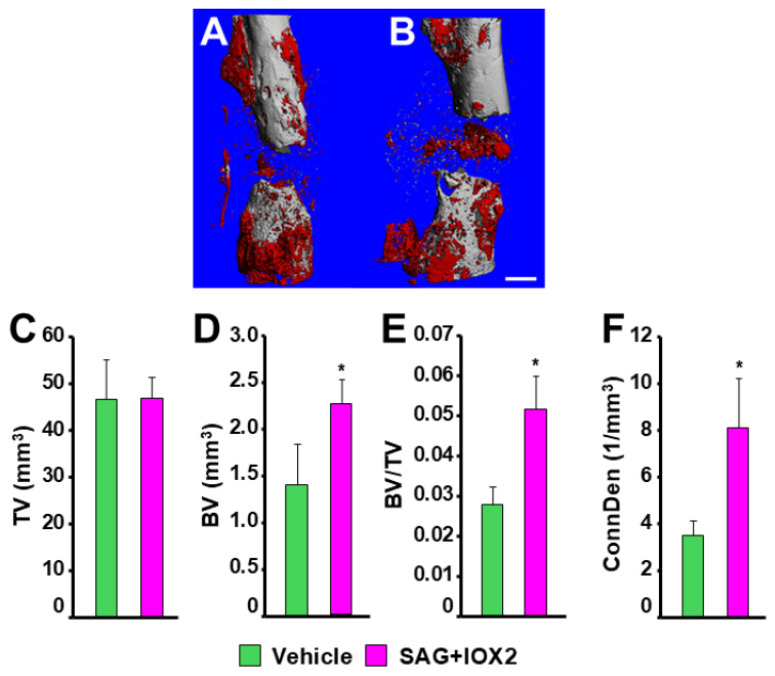
MicroCT 3D analysis of defect callus therapy at 8 weeks post-surgery. In the 3D reconstructions (**A**,**B**), the lower-density woven bone of the defect is colored red for contrast from the higher-density cortical bone. (**A**) Scaffold/BMSC control defect, with limited hard callus bone visible on the cortical bone either side of the defect. (**B**) Scaffold/BMSC/SAG21k-IOX2 therapy, with bone visible as additional hard callus and within the defect. Scale bar = 1 mm. MicroCT measurements quantified the defect callus bone. (**C**) There was no difference in the total volumes of the scaffold/BMSC control and scaffold/BMSC/SAG21k-IOX2 therapy defect calluses. The defect callus bone was increased, as revealed in the (**D**) bone volume (BV), (**E**) the partial bone volume (BV/TV), and (**F**) the connectivity density (Conn Den). * *p* < 0.05.

In vitro microCT analysis confirmed that the therapy did indeed promote bone formation in the defect gap (Figure 7). Specifically, the lower-density bone consistent with defect callus tissue osteogenesis in the vehicle control (Figure 7A) was augmented in response to implants with SAG21k/IOX2 therapy (Figure 7B). The callus volume was not significantly changed (Figure 7C), but measures of bone volume increased significantly in response to the therapy; the increased bone volume (Figure 7D) resulted in an increase in the partial bone volume of the callus (Figure 7E). Additionally, the connectivity density was increased with therapy (Figure 7F), suggesting that the woven bone of the defect callus displayed enhanced trabecular connections consistent with increased bone strength. As observed in the in vivo X-ray controls, there appeared to be less mineralized tissue in the scaffold/BMSC control tissues of the microCT 3D reconstructions and significantly less bone volume, partial bone volume, and connectivity density than in the scaffold/BMSC/SAG21k-IOX2 therapy of the microCT analysis. In the absence of scaffold-alone control, the issue of whether BMSCs contributed substantially to callus bone formation in the absence of osteogenic molecules cannot be determined. The higher-density bone consistent with remodeled callus and cortical bone was not significantly different between applications of therapy and control implants, suggesting that callus bone formation, though augmented, required additional therapy and/or time to complete bony union.

### 3.7. Defect Callus Histology

**Figure 8:**
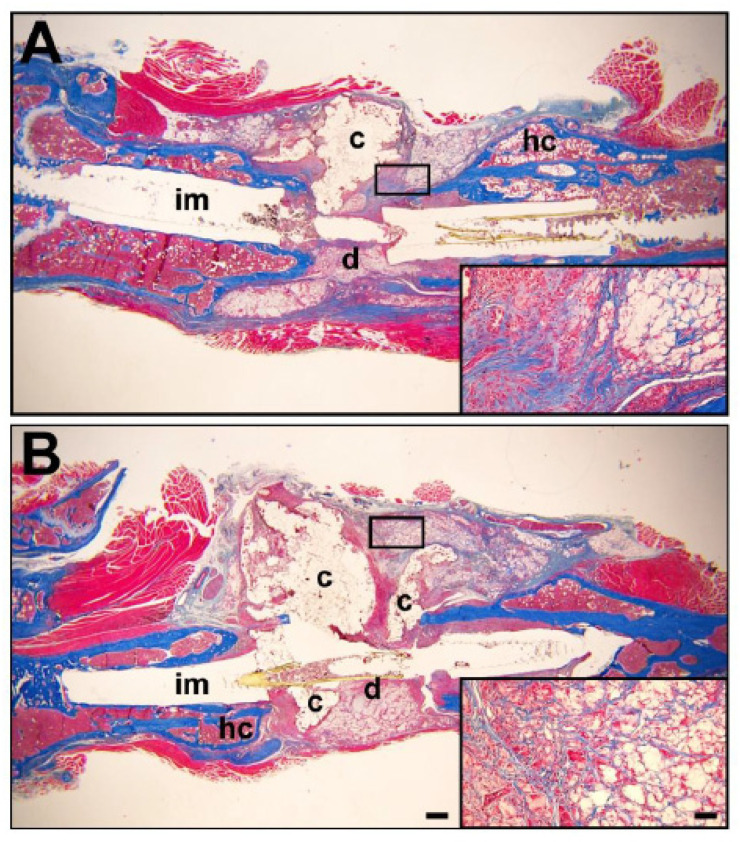
Trichrome histology in segmental defect tissues at 8 weeks post-surgery. Scale bar = 200 μm. Blue staining identifies collagen. Insets identify the high magnifications of defect callus tissues. (**A**) Hard callus is visible at the edges of the scaffold/BMSC control defects, but further bone formation has halted. (**B**) Hard callus is also visible with additional collagen within the implant with scaffold/BMSC/SAG21k-IOX2 therapy. Scale bar = 250 μm in inset. c, scaffold construct; d, defect; hc, hard callus; im, intramedullary space.

Trichrome staining for defect collagen at the eight-week harvest time revealed bone within the defect space. The hard callus that normally develops by intramembranous bone formation early in fracture healing was visible (Figure 8A,B). When compared to the vehicle control tissues (Figure 8A), there appeared to be more soft tissues in response to SAG21k/IOX2 therapy (Figure 8B). The collagen abundance in the defect callus tissue was visible within the matrix of the implant (Figure 8 insets), consistent with increased collagen production representing new bone formation in response to therapy. We admittedly did not characterize endogenous mesenchymal cells for their response to the therapy. A visual examination of the histology did not reveal augmented fat cell content in the defect callus, suggesting that exogenous and endogenous cell differentiation did not augment formation of adipocytes.

## 4. Discussion

The healing of a segmental defect of the long bone is much more problematic than a simple closed fracture. We have developed a segmental defect model of a 2.0 mm femoral critical size defect and previously evaluated sequential small molecule release of SAG21k and IOX2 from an implanted matrix as a therapy for bone formation in a clinically challenging model of bone injury. The success was modest, as despite the support provided by the implant material, the defect size probably presented a distance too great for the development of a viable fracture callus [28]. The space between the ends of the cortical bone is devoid of tissues required for the development of the soft and hard callus tissues or to serve as a support for the cellular regulation of tissue development. We considered whether the inclusion of osteogenic precursor cells that would respond to therapy would promote the development of the callus tissues, using the implant material as a support matrix. BMSCs were chosen as the cell substrate for their well-established osteogenic potential, which would be expected to respond to the therapy.

The fibrin and β-TCP materials chosen for the implant matrix were easily printed in two layers: an outer fibrin layer for the initial burst release of SAG21k to promote chondrogenesis, and an inner β-TCP layer for the subsequent sustained release of IOX2 to promote angiogenesis. The BMSCs were added to the top fibrin layer and the implant wrapped around the rod so that the BMSCs occupied the outer layer and provided a cellular target within the defect, on which the sequential small molecule therapy could promote cartilage and bone formation.

Several studies have applied promoters of osteogenic or angiogenic functions to endochondral segmental defect models of bone healing using implanted scaffolds of biological or synthetic materials, with and without exogenous cells (reviewed in [36,37,38,39]). Autologous bone implants provide an attractive option for healing, although there remain obstacles to their clinical use. Our goal in this study was to test the proof of principle that an implant containing osteogenic stem cells and small molecules that maximize the sequential development of chondrogenesis and osteogenesis would be effective in treating nonunion defects. In our scaffold prototype, the outer fibrin layer not only contained BMSCs but provided an initial burst release of the SMO pathway chondrogenic promoter SAG21k during the dissolution of the fibrin clot and resolution of inflammation in the initial response to the injury. SAG21k therapy is intended to induce the differentiation of osteochondrogenic precursors towards the chondrocyte lineage via activation of sonic hedgehog signaling and augment defect cartilage development. This approach should augment cartilage abundance that is initiated in segmental defect repair but that attenuates before the gap has sufficient tissue to complete the development of a bony fracture callus. However, while canonical cartilage formation is difficult to regulate in fracture repair, enhanced chondrocyte development could also subsequently proceed to osteoblast development and bone formation through transdifferentiation from these chondrocytes [40]. β-TCP was then used to provide a more prolonged release of IOX2 from the inner scaffold layer to increase HIF-1α levels by inhibiting PHD2 activity, an approach to enhance osteogenesis that follows the chondrogenesis promoted by the fibrin-mediated release of SAG21k. IOX2 enhancement of angiogenesis was favored over angiogenic growth factors for this reason; HIF-1α functions are necessary in the hypoxic environment of the defect gap. β-TCP is formulated for strength and a cancellous bone-like porosity [39] that allows for the incorporation of bioactive factors and is one of the more common materials used as a stable matrix for cell support and growth factor delivery [36]. β-TCP seeded with mesenchymal stem cells has been characterized for its bone formation and degradation profiles and was found to provide half-maximal partial bone volume with almost complete degradation by 8 weeks post-implantation [41], characteristics both highly relevant to our application.

BMSCs were chosen as targets for local small molecule therapy for their well-documented chondrogenic response to the hedgehog pathway, and they were utilized for segmental defect chondrogenesis and the osteogenic response in subsequent bone formation [42,43,44]. BMSCs were demonstrated to have good viability in the scaffolds in vitro for a period of 3 weeks. Thus, the implanted cells would be expected to function through the initial critical stages of bone repair, when chondrogenesis promoted by SAG21k would be required for subsequent endochondral bone formation in response to IOX2 enhancement of HIF-1α-mediated angiogenesis. We did not, however, confirm the viability of the BMSCs implanted on the scaffold in the defect in vitro, which might have limited their therapeutic effect in vivo. Additionally, BMSCs responded well to each small molecule in monolayer culture, individually and in combination. SAG21k and IOX2 not only increased expression of genes responsive to hedgehog and hypoxia signaling pathways but also promoted known processes involved in endochondral bone formation, including chondrocyte differentiation, glycolysis, and angiogenesis [45]. Treatments that either did not produce the expected increases in chondrogenic gene expression in response to SAG21k [25] or that produced an increase in *Sox9* and decrease in *Sp7* expression in response to IOX2 suggest that these pathways need further characterization using optimized chondrogenic or osteogenic culture approaches, respectively. These observations suggest that regulation of the sequential release of SAG21k for the development of chondrocytes and IOX2 for the promotion of angiogenesis must be well-regulated temporally to permit chondrogenesis to precede angiogenesis. Accordingly, the immunohistochemistry examination revealed expression of PTCH1 in chondrocytes near the defect edges at 3 weeks post-surgery, when a cartilage formation response would be expected from SAG21k therapy, while HIF-1α expression was observed at the defect site at 8 weeks post-surgery, when bone formation was expected from IOX2 promotion of angiogenesis. These assumptions require further analysis of the cartilage for markers of chondrogenesis and the vasculature for angiogenic markers.

Following surgery, in vivo X-ray analysis of bone formation at the injury site revealed an initial intramembranous hard callus at the cortical surface at the edges of the defect, as observed in closed endochondral bone fracture healing. The appearance of bone within the segmental defect during healing was gradual until the 8-week post-surgery harvest time, and did not appear to follow the callus maturation program in closed fracture healing in which the bony callus eventually closes over the injury to bridge the fracture with mineralized tissue and produce bony union [11]. MicroCT analysis of the defect bone revealed that the lower-density (250–570 mg/cc) bone volume increased compared to the vehicle controls, which in turn increased the partial bone volume (Figure 7), while the higher-density (570–1000 mg/cc) bone was not significantly different between therapy and vehicle treatment. The increased connectivity density in response to therapy did indicate that the mineralized tissue was woven bone, if not a normally developing bony fracture callus. The results of the combined sequential therapy are encouraging, but dose-time studies of the individual SAG21k and IOX2 phases of the sequential therapy must be undertaken to better characterize the segmental defect bone formation.

The histology of the defect callus by 8 weeks post-surgery supported the microCT observations (Figure 8). Trichrome staining for collagen was patchy, while the hard callus that initially developed at the cortical surfaces immediately adjacent to the defect was evident. The implanted construct was visible but appeared to have been reduced in size consistent with its degradation. The collagen visible within the defect matrix indicates that there is the development of tissue within the construct, in addition to bone formation within the defect. Cartilage was very limited by 8 weeks post-surgery, indicating that despite the mechanical stability provided by this segmental defect surgical approach, the BMSCs might have become fibrotic, and chondrocyte development attenuated before complete mineralization of the callus. This aspect of the therapeutic approach requires further study. It is possible that we require a proliferation agent to maximize the cell content of the segmental defect soft callus beyond that of the exogenous BMSCs alone. Importantly, we must account for BMSC contributions to the bone formation effect by comparing the scaffold-alone treatment to a scaffold/BMSC defect treatment.

Several studies have introduced exogenous therapy to endochondral segmental defects, usually as growth factors alone or in combination with implanted autograft or allograft materials. These approaches are designed to recapitulate different aspects of bone repair [37]. In vivo, the bone morphogenetic proteins, (BMPs), notably BMP2 and BMP7, remain the standard to promote bone formation, and have been used alone or in combination. BMP2 has also been used in combination with VEGF to promote angiogenesis and bone formation [46]. Platelet-derived growth factor (PDGF) is thought to mediate bone formation and pericyte mobilization [47] but was not as effective as BMP2 in a rat femoral defect [48]. In these studies, healing is usually evaluated as bone formation after several weeks of healing through radiology and histology approaches. However, comparisons are difficult between the different approaches to experimental segmental defect production, which provide different degrees of stabilization and are often conducted in different animal models. In this respect, our segmental defect model stabilizes the injury similarly to a closed fracture model, promotes bone formation, and allows for comparisons to normal fracture callus endochondral bone formation in similar experimental models. Given sufficient stabilization of the implanted scaffold materials, the application of therapy designed to enhance cartilage and bone formation is possible, as the small molecules and scaffolds proposed for use here are easily available and not toxic in the dosages used for local applications. A requirement for non-immune cells within the scaffolds in patients requires autologous cell isolation, which can be problematic in many clinical settings. The use of cells (biologics) and scaffolds (medical devices) is subject to regulatory requirements by the Food and Drug Administration. Optimizing a sequential therapy application approach should facilitate the development of novel therapy for the repair of nonunion segmental defects for which no treatments are currently available in the clinic [38].

## 5. Conclusions

This therapeutic approach requires refinement of critical variables, but the sequential application of therapeutic agents from a two-layer 3D-printed implant in the presence of cells with chondrogenic and osteogenic capacity has potential for the healing of severe bone injuries. It is obvious that the cell content of the tissues is important for therapeutic functions, because the segmental defect is a gap normally devoid of periosteal cells with chondrogenic and osteogenic potential and susceptible to invasion by non-osteogenic tissues such as muscle [49]. The application of BMSCs with small molecules augmenting the SHH and hypoxia signaling pathways appears to have resulted in partial bone formation within the segmental defect, but this approach was insufficient to enhance the development of a fracture callus capable of bridging the defect gap to provide bony union characteristic of healing within the time period examined.

## Data Availability

All data generated during this study are presented in this article.

## Supplementary Materials

The following supporting information can be downloaded at: https://www.mdpi.com/article/10.3390/biomedicines14010227/s1.
