# Supplementary material for: Enhanced Bone Formation in Segmental Defect Healing Using 3D Printed Scaffolds Containing Bone Marrow Stromal Cells and Small Molecules Targeting Chondrogenesis and Osteogenesis
Source: Biomedicines. 2026 Jan 20;14(1):227. doi: 10.3390/biomedicines14010227 (PMC12838791; doi:10.3390/biomedicines14010227)
Supplement: Supplementary file 1 [file biomedicines-14-00227-s001.zip › biomedicines-3983482-supplementary.pdf]

Table S1. Primer sequences for gene expression.

Gene	Gene Name	Accession	Direction	Sequence
Ki67	Ki67 antigen marker of proliferation	NM_001081117.2	Forward	5’-TGATGTTAGGTGTTTGAGGTCC-3’
			Reverse	5’-CACTTTTCTGGTAACTTCTTGTTC-3’
Ccnd1	Cyclin D1 (v1)	NM_001379248.1	Forward	5’-AATGTACTCTGCTTTGCTGAA-3’
			Reverse	5’-AATGTACTCTGCTTTGCTGAA-3’
Gdp1	Glycerol-3-Phosphate Dehydrogenase 1	NM_010271.3	Forward	5’-AAGTCAAGGGCTGCCGGTT-3’
			Reverse	5’-TCCCTTGGCAGCCTTTGC-3’
Ldha1	Lactate Dehydrogenase A Isoform 1 (v1)	NM_010699.2	Forward	5’-ACGCAGACAAGGAGCAGTGGAA-3’
			Reverse	5’-ATGCTCTCAGCCAAGTCTGCCA-3’
Col2	Collagen 2α1 (v2)	NM_001113515.3	Forward	5’-TGGCTTCCACTTCAGCTATG-3’
			Reverse	5’-AGGTAGGCGATGCTGTTCTT-3’
Acan	Aggrecan	L07049.1	Forward	5’-GACCAGGAAGGGAGGAGTAG-3’
			Reverse	5’-CAGCCGAGAAATGACACC-3’
Col10	Collagen 10α1	NM_009925.4	Forward	5’-ACGGCACGCCTACGATGT-3’
			Reverse	5’-CCATGATTGCACTCCCTG-3’
Mmp13	Matrix Metalloproteinase 13	NM_008607.2	Forward	5’-CATCCATCCCGTGACCTTAT-3’
			Reverse	5’-TCATAACCATTCAGAGCCCA-3’
Sox6	SRY-Box Transcription Factor 6	BC067407.1	Forward	5’-CGGAGGAAGTCGGTGAAGA-3’
			Reverse	5’-GTCGGTTTTGGGAGTGGTG-3’
Sox9	SRY-Box Transcription Factor 9	NM_011448.4	Forward	5’-CGGAGGAAGTCGGTGAAGA-3’
			Reverse	5’-GTCGGTTTTGGGAGTGGTG-3’
Alp	Alkaline Phosphatase (v3)	NM_001429381.1	Forward	5’-ATGGTAACGGGCCTGGCTACA-3’
			Reverse	5’-AGTTCTGCTCATGGACGCCGT-3’
Bsp	Bone Sialoprotein	L20232.1	Forward	5’-AACGGGTTTCAGCAGACAACC-3’
			Reverse	5’-TAAGCTCGGTAAGTGTCGCCA-3’
Bglap/Ocn	Osteocalcin	NM_007541.3	Forward	5’-CTCTCTCTGCTCACTCTGCT-3’
			Reverse	5’-TTTGTAGGCGGTCTTCAAGC-3’
Sp7	Osterix	NM_001348205.1	Forward	5'-TCCTCTCTGCTTGAGGAAGAAG-3'
			Reverse	5'-GAGTCCATTGGTGCTTGAGAAG-3'
Vegf	Vascular Endothelial Growth Factor (v1)	NM_001025257.3	Forward	5’-ATATCAGGCTTTCTGGATTAAGGAC-3’
			Reverse	5’-ATATCAGGCTTTCTGGATTAAGGAC-3’
Gli1	GLI-Kruppel 1	NM_010296.2	Forward	5’-GCACCACATCAACAGTGAGC-3’
			Reverse	5’-GCGTCTTGAGGTTTTCAAGG-3’
Ppia	Peptidylprolyl Isomerase A	NM_008907.2	Forward	5’-CCATGGCAAATGCTGGACCA-3’
			Reverse	5’-TCCTGGACCCAAAACGCTCC-3’

Table S2. Antibody source and application.

Antibody Specificity	Vendor	Catalog #	Dilution	Antigen Retrieval
PTCH1	Novus Biologicals	nb200-118	1:50	UniTrieve Biological
HIF-1α	Novus Biologicals	nb100-134	1:50	UniTrieve Biological
